# Regional Variation in Antenatal Late Preterm Steroid Use Following the ALPS Trial

**DOI:** 10.1001/jamanetworkopen.2023.50830

**Published:** 2024-01-09

**Authors:** Taylor S. Freret, Jessica L. Cohen, Cynthia Gyamfi-Bannerman, Anjali J. Kaimal, Scott A. Lorch, Jason D. Wright, Alexander Melamed, Mark A. Clapp

**Affiliations:** 1 Department of Obstetrics, Gynecology, and Reproductive Biology, Massachusetts General Hospital, Boston; 2 Department of Global Health and Population, Harvard T. H. Chan School of Public Health, Boston, Massachusetts; 3 Department of Obstetrics, Gynecology, and Reproductive Sciences, University of California at San Diego, La Jolla; 4 Department of Obstetrics and Gynecology, University of South Florida, Tampa; 5 Division of Neonatology, Children’s Hospital of Philadelphia, Philadelphia, Pennsylvania; 6 Department of Obstetrics and Gynecology, Columbia University, New York, New York

## Abstract

**Importance:**

The publication of the Antenatal Late Preterm Steroids (ALPS) trial in February 2016 demonstrated that antenatal administration of betamethasone in the late preterm period (between 34 to 36 weeks of gestation) for individuals with a high risk of delivery decreased neonatal respiratory morbidity. National estimates have suggested the trial did change obstetric practice, but little is known if the evidence was adopted uniformly or equitably.

**Objective:**

To assess regional variation in the use of late preterm steroids after the publication of the Antenatal Late Preterm Steroids (ALPS) Trial and to understand factors associated with a region’s pace of adoption.

**Design, Setting, and Participants:**

This cross-sectional study used US natality data from February 2015 to October 2017 from hospital referral regions (HRRs) within the US. Inclusion criteria included live-born, nonanomalous, singleton, late preterm (34 to 36 completed weeks of gestation) neonates born to individuals without pregestational diabetes. This study was conducted from November 15, 2022, to January 13, 2023.

**Main Outcome and Measures:**

HRRs were categorized as either a slower adopter or faster adopter of antenatal late preterm steroids based on the observed vs expected pace of antenatal steroid adoption in a 1-year period after the trial’s dissemination. Patient and regional factors hypothesized a priori to be associated with the uptake of late preterm steroids were compared between faster and slower adopters. Comparisons were made using Student *t* test or Wilcoxon rank-sum test, as appropriate. A multivariable logistic regression was constructed to identify factors associated with faster adopter status in the postperiod.

**Results:**

There were 666 097 late preterm births in 282 HRRs. The mean (SD) maternal age in HRRs was 27.9 (1.2) years. The median (IQR) percentage of births by race categories in HRRs for patients identifying as American Indian or Alaskan Native was 0.5% (0.2%-1.3%); Asian or Pacific Islander, 3.0% (1.7%-5.3%); Black, 12.9% (5.1%-29.1%); and White, 78.6% (66.6%-87.0%). The median percentage of births in HRRs to patients of Hispanic ethnicity was 11.2% (6.3%-27.4%). In this study, 136 HRRs (48.2%) were classified as faster adopters and 146 (51.8%) were classified as slower adopters. Faster adopters increased their steroid use by 12.1 percentage points (from 5.9% to 18.0%) compared with a 5.5 percentage point increase (from 3.7% to 9.2%) among slower adopters (*P* < .001). Most examined patient and regional factors were not associated with a region’s pace of adoption, with the exception of the regional prevalence of prior preterm birth (adjusted odds ratio [aOR], 2.04 [95% CI, 1.48-2.82]) and the percentage of deliveries at 34 to 35 weeks of gestation (aOR, 0.68 [95% CI, 0.47-0.99]) compared with 36 weeks.

**Conclusions and Relevance:**

In this cross-sectional study, there was widespread geographic variation in the adoption of antenatal steroid administration for late preterm births that largely remained unexplained by population factors. These findings should prompt further investigations to barriers to timely or equitable access to new evidence-based practices and guide future dissemination strategies with the goal of more uniform adoption.

## Key Points

**Question:**

Does regional variation exist in the administration of antenatal late preterm steroids, and what factors may be associated with the adoption of this practice?

**Findings:**

In this cross-sectional study of 666 097 late preterm births in 282 hospital referral regions, there was widespread regional variation in the use of late preterm steroids after the publication of the ALPS Trial. Aside from the prevalence of prior preterm birth and distribution of births by gestational age, there were no other identifiable patient or regional characteristics that were associated with the adoption of late preterm steroids.

**Meaning:**

These findings suggest that, despite the dissemination of high-quality evidence and support from obstetric professional societies, antenatal steroid exposure in the late preterm period was not widely adopted, which should inform future studies of the barriers and facilitators of the incorporation of new evidenced-based guidelines into clinical practice.

## Teaser

This cross-sectional study assesses regional variation in the use of late preterm steroids after the publication of the Antenatal Late Preterm Steroids (ALPS) Trial and examines factors associated with a region’s pace of adoption.

## Introduction

The publication of the Antenatal Late Preterm Steroids (ALPS) trial in February 2016 demonstrated that antenatal administration of betamethasone in the late preterm period (between 34 to 36 weeks of gestation) for individuals at high risk of delivery decreased neonatal respiratory morbidity.^1^ Within months following its publication, steroid exposure among infants born in the late preterm period increased by more than 200% nationally.^2^ This practice change was rapid compared with prior studies examining the pace of evidence dissemination in clinical medicine.^3^ However, it is not known if the adoption occurred universally across the US or if specific patient or clinician-level factors may have influenced the use of antenatal steroids in the late preterm period. Therefore, there is a need for more evidence regarding how and why practice changed quickly to better inform policies and care practices around the appropriate and timely use of this intervention.

The primary objective of this study was to examine patterns of regional variation in the administration of antenatal late preterm steroids and determine if regional factors may be associated with the pace of adoption of this practice. Because substantial geographic variation exists in providing other health care services, we hypothesized that there would be measurable variation in late preterm steroid use across hospital referral regions (HRR) in the US and that variation would be attributed to differences in patient case mix.^4,5,6,7^

## Methods

### Study Details

This repeated cross-sectional study was conducted from November 15, 2022, to January 13, 2023, using county-identified US birth certificate data that were obtained with permission from the National Center for Health Statistics.^8^ The project was classified as nonhuman participant research by the Mass General Brigham Human Subjects Research Committee and was exempt from institutional review board approval and informed consent. We followed the Strengthening the Reporting of Observational Studies in Epidemiology (STROBE) reporting guideline.^9^

### Antenatal Steroid Exposure Ascertainment

Antenatal steroid exposure was ascertained from the US birth certificate, which is a standard field included in the 2003 revised version of the US birth certificate and has been used in prior studies examining antenatal steroid use.^2,10,11^ The exposure is reported as a binary variable (yes or no) and does not capture details on the gestational age at which the steroids were administered.

### ALPS Trial Dissemination and Analysis Periods

The ALPS trial was first available online in February 2016, published in print in April 2016, and included in updated clinical guidance in August and October 2016 from the Society for Maternal-Fetal Medicine (SMFM) and the American College of Obstetricians and Gynecologists (ACOG), respectively.^1,12,13,14^ Thus, we considered February to October 2016 to be the period of evidence dissemination, as designated in previous work.^2^ Data from a 12-month observational period before (ie, preperiod from February 2015 to January 2016) and after (ie, postperiod from November 2016 to October 2017) the dissemination period were used to assess variation in late preterm steroid use and the pace of adoption.

### Unit of Analysis

Because of the small number of preterm births in many counties, geographic analysis was performed at the hospital referral region (HRR) level. HRRs were defined by the Dartmouth Atlas Project and represent regional health care markets for tertiary medical care.^15^ County-level federal information processing system (FIPS) codes from delivery locations were mapped to HRRs using previously published crosswalks.^15^

To facilitate meaningful comparisons across the time periods and reduce noise in the estimates of the expected steroid use in the postperiod, 2 HRR inclusion criteria were set: (1) more than 100 eligible late preterm births in the preperiod and (2) steroid exposure data available for all eligible births. Eligible births were defined as liveborn, nonanomalous singleton neonates born between 34 and 36 completed weeks of gestation to individuals without pregestational diabetes (ie, participants most similar to those in the ALPS trial).^2^ Births were not eligible for inclusion if antenatal steroid exposure was unknown, if data elements were not reliably reported per the Natality Data User Guides, or if the FIPS code for the location in which they delivered was unknown.^16^

### Statistical Analysis

#### Defining Pace of Adoption

The primary outcome of interest was the HRR-level adoption status of late preterm steroid use, designated as either slower or faster adopters. Adoption status was assigned by comparing the observed rates of late preterm births exposed to antenatal steroids compared with the expected rates of steroid exposure in the 12-month period following the ALPS trial dissemination. Expected rates were estimated from a patient-level logistic regression model that included a fixed effect for the HRR and patient factors hypothesized a priori to influence adoption (ie, factors that may inform counseling or influence a clinician’s estimation of a patient’s risk of late preterm birth). These factors included maternal age (continuous), parity (categorized as nulliparous or multiparous), race (precategorized in the US natality data as American Indian or Alaskan Native, Asian or Pacific Islander, Black, or White), ethnicity (precategorized as Hispanic or non-Hispanic in the US natality data), primary payer for delivery (categorized as private, public, self-pay, or other), education (binary variable indicating some postsecondary education), maternal comorbidities (binary variables indicating the presence or absence of pregnancy-related and preexisting hypertensive disorders and gestational diabetes), week of gestation at delivery (categorical), an indicator if a maternal transfer occurred before delivery, and an indicator if the patient delivered in the same county as their residential address. Race and ethnicity were included not based on a biological hypothesis but because preexisting literature has suggested rates of steroid administration vary across these groups.^17^ As an underlying increasing trend in antenatal steroid exposure was observed in the preperiod, which is hypothesized to exist due to local changes in birth certificate reporting practices, the regression model also adjusted for preperiod steroid exposure trends in each HRR (determined by including a categorical variable for each HRR and an interaction term between HRR and month in the preperiod in the model). The model used robust standard errors to account for HRR clustering. The preperiod multivariable regression model results are included in eTable 1 in Supplement 1.

Coefficients from the regression model derived from the preperiod data were applied to the postperiod data to generate a probability of steroid receipt for each eligible patient. These estimated probabilities were summed within each HRR to determine the expected number of patients who received steroids and the steroid administration rate in the postperiod. The difference in the observed and expected rates of steroid exposure in the postperiod was calculated for each HRR. Rate differences less than the median were considered slower adopters; HRRs with rate differences equal to or greater than the median were considered faster adopters.

#### Factors Associated With Adopter Status

Once adopter status had been assigned, we examined regional factors associated with the adoption pace. The following HRR population characteristics were compared between slower and faster adopters at the HRR level in the preperiod: mean maternal age, race (American Indian or Alaskan Native, Asian or Pacific Islander, Black, or White, using the maternal bridged race categories within the US natality data), Hispanic ethnicity, education, gestational diabetes, gestational hypertension, chronic hypertension, prior preterm birth, and primary payer for delivery admission. Except for maternal age (which used population means), all variables were expressed as a percentage of late preterm births. Similarly, the following HRR regional characteristics were compared between slower and faster adopters in the preperiod: delivery clinician (percentage of late preterm births attended by a physician, midwife, or other), rate of infant transfers, number of hospitals with obstetric care per square mile, number of births per obstetric bed, number of births per higher-level pediatric bed (neonatal intensive care unit or special care nursery), population density, and total geographic area (square mile). Variables related to the number of hospitals, beds, births, and geographic area were obtained from the 2017 Area Health Resource Files, publicly available from the US Health Resources and Services Administration.^18^ These covariates were selected a priori based on known associations with preterm birth or hypothesized to be related to an area’s tendency to adopt late preterm steroids into practice.^19,20,21,22^ Comparisons were made using Student *t* test or Wilcoxon rank-sum test, as appropriate. Then, a multivariable logistic regression was constructed using the aforementioned covariates to identify factors associated with faster adopter status in the postperiod. To facilitate the comparison of the coefficients, standardized mean differences were used for variables that reflected population percentages.

Analyses were performed using Stata MP software, version 15.1 (StataCorp). An a priori 2-sided *P* <.05 was considered statistically significant. Statistical analyses were performed from November 15, 2022, to January 13, 2023.

#### Sensitivity Analysis

The adoption status was defined for the primary analysis based on the median rate difference. As sensitivity analyses, we also performed the multivariable regression model to identify factors associated with adopter status based on the highest and lowest quartile of rate differences; this approach allowed for more distinction in the rate differences between the 2 groups. As a separate analysis, we examined the association between the same factors associated with an HRR’s observed vs expected rate difference (continuous outcome) using linear regression rather than dichotomizing it into slower or faster adopter groups. This analysis allowed for a more granular analysis of factors influencing the change in steroid use, though it did not allow for a comparison of an HRR’s adoption pace relative to other HRRs.

## Results

There are 306 HRRs in the United States. Twenty-four were excluded: 12 could not be crosslinked to county-level zip codes, and 12 had fewer than 100 births recorded in the preperiod. From the 282 included HRRs, a total of 666 097 late preterm births occurred between February 1, 2015, and October 31, 2017. Of these births, 239 372 (35.9%) occurred in the preperiod, 182 352 (27.4%) in the dissemination period, and 244 373 (36.7%) in the postperiod. The mean (SD) maternal age in HRRs was 27.9 (1.2) years. The median (IQR) percentage of births by race categories in HRRs for patients identifying as American Indian or Alaskan Native was 0.5% (0.2%-1.3%); Asian or Pacific Islander, 3.0% (1.7%-5.3%); Black, 12.9% (5.1%-29.1%); and White, 78.6% (66.6%-87.0%). The median percentage of births in HRRs to patients of Hispanic ethnicity was 11.2% (6.3%-27.4%).

**Figure 1.**
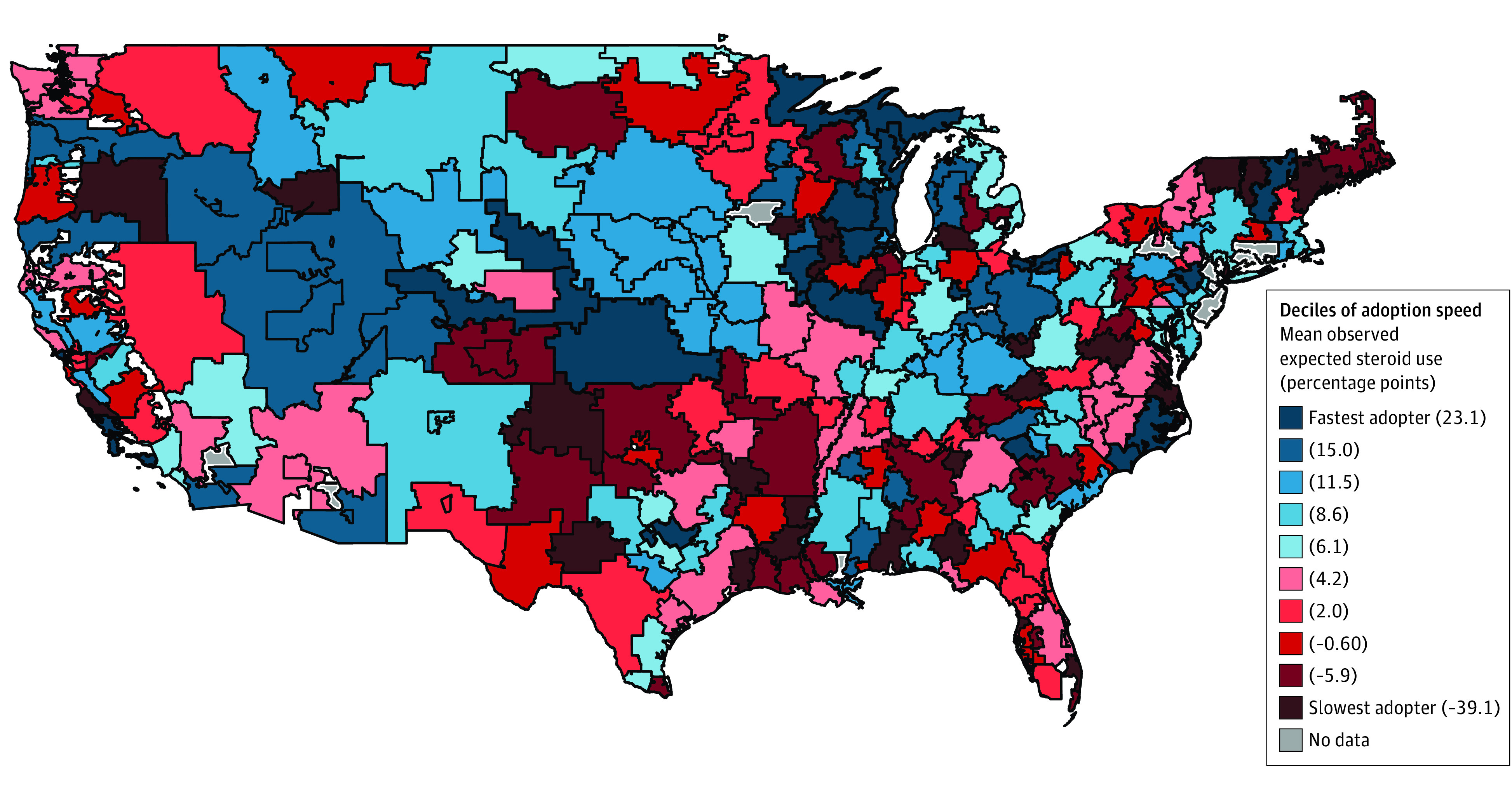
Heat Map of Late Preterm Steroid Adoption After the Antenatal Late Preterm Steroid Trial by Hospital Referral Region Adopter status determined by the difference between observed and expected steroid use in the postperiod relative to the median change among all hospital referral region in the year following the Antenatal Late Preterm Steroid Trial.

**Figure 2.**
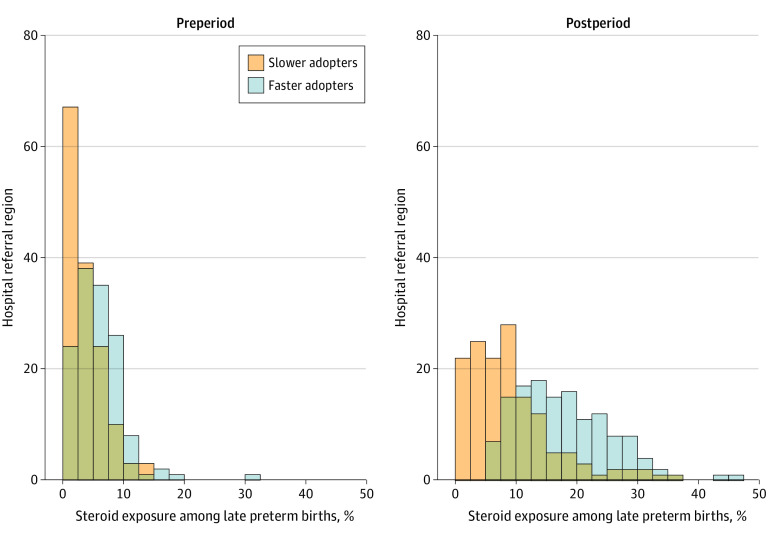
Histogram of Antenatal Late Preterm Steroid Use in the Pre- and Postperiods by Hospital Referral Region (HRR) Adopter Status In the year before the Antenatal Late Preterm Steroid Trial (preperiod), there was similar late preterm steroid use between the slower and faster adopter HRRs. In the year following the trial (postperiod), the use diverged between the 2 groups. Orange indicates slower adopters and blue indicates faster adopters.

After the publication of the ALPS Trial, late preterm steroid use among HRRs ranged from 0 to 47.4% with median of 11.7% (IQR, 6.9% to 18.6%). There was widespread geographic variation noted relative to the expected steroid rate in the postperiod, with differences in the observed and expected rates ranging from 94.8% less than expected to 33.8% more than expected based on patient characteristics and preperiod trends (median, 4.9% [IQR, −0.8% to 11.0%]) more than expected (Figure 1). Of the 282 HRRs, 136 (48.2%) were designated as faster adopters and 146 (51.8%) as slower adopters. In HRRs categorized as faster adopters, steroid use increased from 5.9% to 18.0% between the pre- and postperiods. In comparison, in slower adopting HRRs, there was a 5.5 percentage point increase in steroid use from 3.7% to 9.2% throughout the same period. This corresponded to a 12.1 percentage point increase (from 5.9% to 18.0%) in steroid-exposed births after the dissemination period in HRRs designated as faster adopters (*P* < .001) (Figure 2).

Baseline characteristics between faster and slower adopters were similar in the preperiod (Table 1). Three characteristics were notably different: faster adopters had a lower median (IQR) proportion of patients with some postsecondary education experience (45.9% [40.5%-51.2%] vs 49.6% [43.0%-55.0%]; *P* = .001), a higher proportion of deliveries covered by private insurance (43.3% [34.8%-51.3%] vs 37.2% [31.2%-46.0%]; *P* < .001), and a higher mean (SD) rate of patients with a prior preterm birth (9.1% [6.3%-12.4%] vs. 7.6% [4.0%-10.1%]; *P* < .001) compared with slower adopter regions.

**Table 1.** Baseline Characteristics of Hospital Referral Regions (HRRs) in the Preperiod

Characteristics	Patient, mean (SD), %		*P* value
	Slower adopters, (n = 118 364)	Faster adopters, (n = 121 008)	
Total HRRs, No.	146	136	NA
Population characteristics			
Maternal age, y	27.7 (1.2)	28.2 (1.3)	.004
Race and ethnicity^a^			
American Indian or Alaskan Native	0.5 (0.1-1.3)	0.4 (0.2-1.3)	.47
Asian or Pacific Islander	2.7 (1.7-4.9)	3.5 (1.8-5.8)	.09
Black	14.4 (5.4-28.2)	11.5 (4.6-24.1)	.26
Hispanic^a^	10.9 (6.1-27.0)	12.1 (6.4-27.4)	.80
White	78.1 (66.2-87.0)	79.3 (69.6-86.7)	.68
Any postsecondary education^a^	49.6 (43.0-55.0)	45.9 (40.5-51.2)	.001
Gestational diabetes^a^	7.4 (5.6-9.4)	8.4 (6.6-9.8)	.01
Gestational hypertension^a^	10.7 (8.0-12.8)	11.7 (9.3-13.8)	.02
Chronic hypertension^a^	2.6 (1.6-3.7)	2.9 (1.9-4.2)	.06
Prior preterm birth^a^	7.6 (4.0-10.1)	9.1 (6.3-12.4)	<.001
Insurance^a^			
Private	37.2 (31.2-46.0)	43.3 (34.8-51.3)	<.001
Public	53.8 (45.3-61.5)	48.7 (41.3-58.3)	.004
Self-Pay	2.7 (1.5-4.9)	2.5 (1.4-4.2)	.51
Other	1.9 (0.6-5.1)	2.4 (0.9-4.5)	.67
Health system characteristics			
Gestational age at delivery, wk^a^			
34	17.7 (16.1-19.2)	17.9 (16.4-19.2)	.34
35	28.6 (27.4-29.7)	27.9 (26.5-29.6)	.04
36	54.1 (51.7-56.1)	54.1 (51.8-55.9)	.93
Delivery clinician^a^			
MD/DO	93.2 (87.5-97.1)	93.3 (89.4-96.9)	.57
CNM	5.8 (2.0-10.6)	5.4 (2.3-9.5)	.88
Other	0.8 (0.3-1.7)	0.6 (0.3-1.2)	.16
Infant transfer^a^	2.0 (0.9-3.9)	2.2 (1.1-3.5)	.96
OB hospital density, /100 mi^2^^b^	0.6 (0.3-1.1)	0.7 (0.4-1.7)	.02
Total births per OB bed^c^	70.8 (56.5-91.8)	70.1 (58.7-87.6)	.85
Total births per higher-level pediatric bed^d^	155.0 (113.1-212.1)	145.9 (117.6-202.1)	.91
Geographic characteristics			
Population density, /100 mi^2^	330.4 (144.8-687.3)	344.9 (180.7-936.2)	.29
Total area, mi^2^	863.0 (573.5-1423.8)	760.1 (535.8-1264.8)	.25

Abbreviations: CNM, certified nurse-midwife; DO, Doctor of Osteopathic Medicine; MD, medical doctor; NA, not applicable; OB, obstetric.

^a^ Listed as a percentage of the HRR population.

^b^ OB hospital density refers to the number of hospitals with obstetric care, as defined by the 2017 Area Health Resources File per total HRR land area.

^c^ Total births refer to the total number of births in the HRR (including all preterm and term births).

^d^ Higher-level pediatric beds include neonatal intensive care units and special care nursery beds.

In the multivariable logistic regression analysis, the percentage of the population with a prior preterm birth was significantly associated with faster adopter status (adjusted odds ratio [aOR], 2.04; 95% CI, 1.48-2.82) (Table 2). This coefficient can be interpreted as the change in odds associated with a 1 standardized mean difference increase in the population rate of prior preterm birth. Also, compared with the percentage of births at 36 weeks of gestation in an HRR, those with higher percentages of births between 34 to 35 weeks of gestation were less likely associated with adopter status (aOR, 0.68; 95% CI, 0.47-0.99) (Table 2). In the sensitivity analyses using the alternate definition for HRR adopter status (top vs bottom quartile), only the percentage of the patients who had prior preterm birth was significantly associated with being a faster adopter (aOR, 1.83; 95% CI, 1.21-2.78) (eTable 2 in Supplement 1).

**Table 2.** Multivariable Estimators of Hospital Referral Regions Late Preterm Steroid Adopter Status^a^

Characteristics	Adjusted OR (95% CI)	*P* value
Population characteristics		
Maternal age	0.95 (0.60-1.52)	.83
Race		
White	1 [Reference]	.78
Other^b^	0.95 (0.65-1.38)	
Hispanic ethnicity	1.12 (0.77-1.65)	.55
Any postsecondary education	0.74 (0.47-1.16)	.19
Gestational diabetes	1.01 (0.72-1.41)	.96
Gestational hypertension	1.14 (0.84-1.53)	.40
Chronic hypertension	1.32 (0.93-1.86)	.12
Prior preterm birth	2.04 (1.48-2.82)	<.001
Insurance		
Private	1 [Reference]	.62
Nonprivate	1.13 (0.69-1.86)	
Health system characteristics		
Gestational age at delivery, wk		
34-35	0.68 (0.47-0.99)	.04
36	1 [Reference]	
Delivery clinician		
Doctor	1 [Reference]	.53
Midwife or other	1.09 (0.83-1.44)	
Infant transfer	0.87 (0.62-1.20)	.39
OB hospital density, /100 mi^2^	1.10 (0.84-1.44)	.49
Total births (hundreds) per OB bed	1.67 (0.65-4.32)	.29
Total births (hundreds) per higher-level pediatric bed	0.88 (0.73-1.07)	.21
Geographic characteristics		
Population density, /100 mi^2^	1.04 (0.99-1.10)	.08
Total area, /100 mi^2^	1.00 (0.98-1.02)	.76

Abbreviations: NA, not applicable; OB, obstetric; OR, odds ratio.

^a^ Regression estimating faster adopter status includes all variables listed. As the analysis was performed at the regional (not patient) level, characteristics are expressed as a percentage of the population. When the underlying patient-level variable was categorical (race, insurance, gestational at delivery, delivery clinician), the most frequent response (ie, population majority) was compared with the other groups combined in this regional-level analysis. This approach is limited because it precludes a more granular assessment of the grouped categories and assumes that the grouped categories have a similar association with the comparator.

^b^ Includes participants identifying as American Indian or Alaskan Native, Asian or Pacific Islander, or Black.

eTable 3 in Supplement 1 demonstrates the following HRR characteristics that were significantly associated with the continuous observed vs expected rate difference (expressed as percentage points), rather than adopter status. The adjusted regression coefficient for the mean maternal age was 0.038 (95% CI, 0.005 to 0.070); prevalence of gestational hypertension, 0.029 (95% CI, 0.007 to 0.051); prevalence of prior preterm birth, 0.034 (95% CI, 0.012 to 0.056); percentage of deliveries at 34 to 35 weeks of gestation vs 36 weeks of gestation, −0.031 (95% CI, −0.052 to −0.010); and total number of births per higher-level pediatric beds (ie, neonatal intensive care or intermediate care), −0.012 (95% CI, −0.021 to −0.002).

## Discussion

The ALPS Trial demonstrated that antenatal steroid administration during the late preterm period reduced neonatal respiratory morbidity, a major cause of health care resource use in the US, with a secondary analysis demonstrating that late preterm steroids are likely cost-effective.^23^ These findings have also been shown outside of a clinical trial setting in a subsequent study.^1,2^ Professional societies in the United States (namely, ACOG and SMFM) have recommended late preterm steroids in eligible populations. Despite these recommendations, we observed significant regional variation in adopting this practice across the US and overall high levels of nonadoption. This nonuniform uptake raises additional questions about why this multicenter randomized trial and nationwide dissemination strategy did not result in a more uniform or anticipated widespread uptake and what additional data or information may be needed to influence adoption.

Among patient factors, the population prevalence of prior preterm birth was associated with ALPS adoption. The ALPS trial highlighted the challenge of appropriately identifying who is at risk for preterm birth, with nearly 20% of the trial participants ultimately delivering at full term.^1^ As prior preterm birth is a strong factor for recurrent preterm birth, we hypothesize that in areas where the a priori risk of preterm birth may be higher (eg, more individuals with a prior preterm birth), clinicians may be more likely to recommend and administer late preterm steroids and that health systems may be better equipped to administer late preterm steroids due to increased underlying need. In contrast, the population prevalence of hypertensive disorders of pregnancy, a common medical indication for a late preterm birth, was not associated with ALPS adoption. However, the ascertainment of the timing and severity of this condition is not well known from the natality data and should continue to be explored.

Of note, we did observe that the proportion of deliveries at 34 to 35 weeks of gestation relative to 36 weeks was weakly associated with lower odds of being a faster adopter (*P* = .04). This association was not present in the sensitivity analysis defining adopter status using the top and bottom quartile, lessening the likelihood of a significant impact. However, we did observe a similar negative association with an increasing rate difference (ie, higher steroid use than was expected) for HRRs with a higher proportion of deliveries at 34 to 35 weeks compared with 36 weeks. This finding may reflect an underlying limitation of the dataset because only the week of gestation of delivery and if antenatal steroids were administered during the preterm period were known, while the exact gestational age of administration was unknown.

Because other regional characteristics, such as region size, population density, obstetric, and neonatal bed availability, were not associated with the pace of adoption, late preterm steroid use may be associated with more granular clinician- and hospital-level practices and patient preferences, many of which cannot be measured in population-based data sources. Future quantitative and qualitative work should seek to identify more local sources of practice variation and identify barriers to adoption. These results will inform policies to guide the timely and appropriate use of late preterm steroids and the dissemination of new evidence-based medicine practices in obstetrics.

The strengths of this study include the use of US natality data, which represents a complete sample of late preterm births in the US, and method incorporating preexisting time trends (in addition to fixed factors) in each HRR to estimate the expected steroid exposure in the postperiod. The natality data are one of the only sources where antenatal steroid exposure, birth outcomes, and maternal information can be ascertained.

### Limitations

This study has limitations. HRRs were not constructed around perinatal or obstetric care services specifically, though they are commonly used to examine regional practice variation in other areas of medicine. As each HRR encompasses many counties, the case mix, health systems, and geographic characteristics represent averages expected to trend toward the population mean, which may limit the ability to discern the impact of any specific characteristic. Additionally, we could not control for factors that may have influenced the clinical or shared decision-making around steroid administration, such as the indication or likelihood of a late preterm delivery. The exact timing of steroid administration is unknown in the natality data.

Certain elements of birth certificate data may be subject to low sensitivity or specificity, including steroid administration; steroid administration rates are likely underreported but not systematically in a way that negates the study findings.^24^ We accounted for potentially inaccurate reporting by excluding births with concerns regarding data reliability per the yearly Natality Data User Guides. It is also possible that birth certificate reporting practices varied between each HRR over time, in which case the relative changes of the observed and expected rates of steroid exposure may not have been uniform. We accounted for this potential variation by adjusting the models for preexisting trends in steroid use in each HRR; however, we cannot estimate or observe which HRRs may have differentially increased reporting after the ALPS Trial publication. Finally, the US birth certificate does not capture the timing of antenatal steroid exposure, only whether the steroids were administered at any point in the pregnancy; thus, this study captures late preterm births exposed to antenatal steroids (at any point in pregnancy). Ideally, we would study antenatal steroid administration during the late preterm period; however, there is no US population-based data set to conduct a study of this nature.

## Conclusion

In this cross-sectional study of HRRs, there was widespread regional variation in the use of late preterm steroids after the publication of the ALPS Trial. Aside from preterm birth prevalence and gestational age distribution, there were no other identifiable patient or regional characteristics associated with the adoption of late preterm steroids in the primary model. This study highlights how, despite the dissemination of high-quality evidence and support from obstetric professional societies, antenatal steroid exposure in the late preterm period was not uniformly adopted, which should inform future studies on the barriers and facilitators of the incorporation of new evidence-based guidelines into clinical practice.

## Notes

Supplement 1. **eTable 1.** Coefficients for Multivariable Model to Estimate Expected Late Preterm Steroid Exposure in the Postperiod **eTable 2.** HRR Characteristics Associated With Being a Faster Adopter Compared With a Slower Adopter Using Alternate Definition **eTable 3.** HRR Characteristics Associated With the Observed vs Expected Steroid Rate Difference in the Postperiod

Supplement 2.
Data Sharing Statement
