# Supplementary material for: Regional Variation in Antenatal Late Preterm Steroid Use Following the ALPS Trial
Source: JAMA Netw Open. 2024 Jan 9;7(1):e2350830. doi: 10.1001/jamanetworkopen.2023.50830 (PMC10777258; doi:10.1001/jamanetworkopen.2023.50830)
Supplement: Supplement 1. — eTable 1. Coefficients for Multivariable Model to Estimate Expected Late Preterm Steroid Exposure in the Postperiod eTable 2. HRR Characteristics Associated With Being a Faster Adopter Compared With a Slower Adopter Using Alternate Definition eTable 3. HRR Characteristics Associated With the Observed vs Expected Steroid Rate Difference in the Postperiod [file jamanetwopen-e2350830-s001.pdf]

Supplemental Online Content

Freret TS, Cohen JL, Gyamfi-Bannerman C, et al. Regional variation in antenatal late preterm steroid use following the ALPS trial. *JAMA Netw Open*. 2023;7(1):e2350830. doi:10.1001/jamanetworkopen.2023.50830

eTable 1. Coefficients for Multivariable Model to Estimate Expected Late Preterm Steroid Exposure in the Postperiod
eTable 2. HRR Characteristics Associated With Being a Faster Adopter Compared With a Slower Adopter Using Alternate Definition
eTable 3. HRR Characteristics Associated With the Observed vs Expected Steroid Rate Difference in the Postperiod

This supplemental material has been provided by the authors to give readers additional information about their work.

© 2024 Freret TS et al. *JAMA Network Open.*

eTable 1. Coefficients for Multivariable Model to Estimate Expected Late Preterm Steroid Exposure in the Postperiod

Model coefficients derived from preperiod data (ie, before the publication of the ALPS Trial) and then used to predict the patient-level probability of steroid exposure in the post-period (i.e., after dissemination of the ALPS Trial).

Characteristic	Adjusted Odds Ratio	p-value
Month	1.15 [1.15-1.15]	<0.001
Maternal Age	1.00 [0.99 – 1.00]	0.47
Parity		
Nulliparous	Reference	
Multiparous – No prior preterm birth	0.83 [0.79 - 0.88]	<0.001
Multiparous – Prior preterm birth	2.46 [2.27 - 2.66]	<0.001
Week of Gestation		
34 Weeks	Reference	
35 Weeks	0.39 [0.37 - 0.41]	<0.001
36 Weeks	0.20 [0.19 - 0.22]	<0.001
Gestational Diabetes	1.25 [1.16 - 1.35]	<0.001
Gestational Hypertension	1.63 [1.54 - 1.74]	<0.001
Chronic Hypertension	1.67 [1.51 - 1.86]	<0.001
Provider Type		
Physician	Reference	
Midwife	0.51 [0.43 - 0.60]	<0.001
Other	0.53 [0.39 - 0.31]	<0.001
Payer		
Medicaid	Reference	
Private	1.06 [0.99 - 1.13]	0.07
Self-pay	0.76 [0.64 - 0.91]	0.002
Other	0.91 [0.78 - 1.07]	0.26
Delivery Location in Same County as Residence	0.80 [0.75 - 0.85]	<0.001
Maternal Transfer	3.06 [2.77 - 3.50]	<0.001
Maternal Education		
Did Not Complete High School	Reference	
High School Degree	1.13 [1.06 - 1.22]	<0.001
Some College / Associate's Degree	1.24 [1.15 - 1.32]	<0.001
Bachelor's Degree	1.22 [1.11 - 1.34]	<0.001
Graduate Degree	1.34 [1.20 - 1.50]	<0.001
Ethnicity		
Non- Hispanic	Reference	
Hispanic	0.90 [0.84 - 0.97]	0.003
Race		
Asian or Pacific Islander	0.84 [0.76 - 0.94]	0.002
Black	0.95 [0.86 - 1.04]	0.29
White	Reference	
American Indian or Alaskan Native	0.63 [0.51 - 0.78]	<0.001
Fixed Effect of Each HRR	**not shown given number of HRR's*	

© 2024 Freret TS et al. *JAMA Network Open.*

eTable 2: HRR Characteristics Associated with Being a Faster Adopter Compared With a Slower Adopter Using Alternate Definition*

* Alternate definition for adoption status: faster adopters are defined using the top quartile of the observed to expected rate difference, slower adopters as bottom quartile.

**Population Characteristics**	**Adjusted Odds Ratio**	**p-value**
Maternal age	1.19 [0.62 - 2.25]	0.60
Race[†]		
White	Reference	
Other[a]	0.64 [0.36 - 1.14]	0.13
Hispanic Ethnicity	1.02 [0.59 - 1.76]	0.95
Any Postsecondary Education	0.72 [0.37 - 1.42]	0.35
Gestational Diabetes	1.32 [0.83 - 2.10]	0.24
Gestational Hypertension	1.28 [0.85 - 1.94]	0.23
Chronic Hypertension	1.65 [1.00 - 2.73]	0.05
Prior Preterm Birth	1.83 [1.21 - 2.78]	<0.001
Insurance		
Private	Reference	
Non-Private	1.17 [0.59 - 2.33]	0.65
**Health System Characteristics**		
Gestational Age at Delivery		
34-35 Weeks	0.95 [0.59 - 1.54]	0.84
36 Weeks	Reference	
Delivery Provider		
Doctor	Reference	
Midwife/Other	0.82 [0.54 - 1.25]	0.35
Infant Transfer	0.87 [0.57 - 1.32]	0.51
OB Hospital Density (per 100 sq. mi)	1.42 [0.90 - 2.24]	0.14
Total Births (Hundreds) per OB Bed	0.83 [0.21 - 3.24]	0.79
Total Births (Hundreds) per Higher-level Pediatric Bed	0.95 [0.77 - 1.16]	0.59
**Geographic Characteristics**		
Population Density (in 100s per sq. mi)	1.04 [0.96 - 1.12]	0.33
Total Area (100 sq. mi)	1.04 [0.99 - 1.08]	0.10

[a]Includes participants identifying as American Indian or Alaskan Native, Asian or Pacific Islander, or Black.
The logistic regression predicting faster adopter status includes all variables listed. As the analysis was performed at the regional (not patient) level, characteristics are expressed as percent of the population. When the underlying patient-level variable was categorical (race, insurance, gestational at delivery, delivery provider), the most frequent response (i.e., population majority) was compared to the other groups combined in this regional-level analysis. We acknowledge that a limitation of this approach is that it precludes a more granular assessment of the grouped categories and assumes that the grouped categories have a similar relationship with the comparator.

© 2024 Freret TS et al. *JAMA Network Open.*

eTable 3. HRR Characteristics Associated With the Observed vs Expected Steroid Rate Difference in the Postperiod

Rate difference expressed in terms of percentage points. Higher positive rate differences corresponded to higher-than-expected levels of adoption.

**Population Characteristics**	**Adjusted Coefficient**	**p-value**
Maternal age (years)	0.038 [0.005 - 0.070]	0.02
Race†		
White	Reference	
Other[a]	0.000 [-0.027 - 0.027]	1.00
Hispanic Ethnicity	0.007 [-0.021 - 0.035]	0.61
Any Postsecondary Education	-0.006 [-0.039 - 0.027]	0.72
Gestational Diabetes	-0.002 [-0.026 - 0.023]	0.90
Gestational Hypertension	0.029 [0.007 - 0.051]	0.01
Chronic Hypertension	0.019 [-0.006 - 0.044]	0.14
Prior Preterm Birth	0.034 [0.012 - 0.056]	<0.001
Insurance		
Private	Reference	
Non-Private	0.023 [-0.015 - 0.06]	0.23
**Health System Characteristics**		
Gestational Age at Delivery		
34-35 Weeks	-0.031 [-0.052 - -0.010]	<0.001
36 Weeks	Reference	
Delivery Provider		
Doctor	Reference	
Midwife/Other	0.006 [-0.015 - 0.026]	0.59
Infant Transfer	0.012 [-0.012 - 0.036]	0.31
OB Hospital Density (per 100 sq. mi)	0.009 [-0.009 - 0.026]	0.32
Total Births (Hundreds) per OB Bed	0.008 [-0.057 - 0.073]	0.80
Total Births (Hundreds) per Higher-level Pediatric Bed	-0.012 [-0.021 - -0.002]	0.02
**Geographic Characteristics**		
Population Density (in 100s per sq. mi)	0.000 [-0.001 - 0.001]	0.41
Total Area (100 sq. mi)	0.001 [-0.001 - 0.002]	0.31

[a]Includes participants identifying as American Indian or Alaskan Native, Asian or Pacific Islander, or Black.

The linear regression model predicting rate difference includes all variables listed. As the analysis was performed at the regional (not patient) level, characteristics are expressed as percent of the population. When the underlying patient-level variable was categorical (race, insurance, gestational at delivery, delivery provider), the most frequent response (i.e., population majority) was compared to the other groups combined in this regional-level analysis. We acknowledge that a limitation of this approach is that it precludes a more granular assessment of the grouped categories and assumes that the grouped categories have a similar relationship with the comparator.

© 2024 Freret TS et al. *JAMA Network Open.*
