# Supplementary material for: Regional Variation in Antenatal Late Preterm Steroid Use Following the ALPS Trial
Source: JAMA Netw Open. 2024 Jan 9;7(1):e2350830. doi: 10.1001/jamanetworkopen.2023.50830 (PMC10777258; doi:10.1001/jamanetworkopen.2023.50830)
Supplement: Supplement 2. — Data Sharing Statement [file jamanetwopen-e2350830-s002.pdf]

Data Sharing Statement

Freret. Regional Variation in Antenatal Late Preterm Steroid Use Following the ALPS Trial. *JAMA Netw Open*. Published January 09, 2024. doi:10.1001/jamanetworkopen.2023.50830

Data
Data available: No
